# Sugarcane Ratooning Ability: Research Status, Shortcomings, and Prospects

**DOI:** 10.3390/biology10101052

**Published:** 2021-10-15

**Authors:** Fu Xu, Zhoutao Wang, Guilong Lu, Rensen Zeng, Youxiong Que

**Affiliations:** 1 Key Laboratory of Sugarcane Biology and Genetic Breeding, Ministry of Agriculture and Rural Affairs, Fujian Agriculture and Forestry University, Fuzhou 350002, China; 2200101003@fafu.edu.cn (F.X.); 2170102010@fafu.edu.cn (Z.W.); 2190102004@fafu.edu.cn (G.L.); 2 Key Laboratory of Ministry of Education for Genetics, Breeding and Multiple Utilization of Crops, College of Agriculture, Fujian Agriculture and Forestry University, Fuzhou 350002, China; rszeng@fafu.edu.cn

**Keywords:** sugarcane ratooning ability, trait markers, sugarcane breeding, sugar production, segregating population, sugarcane stubble

## Abstract

**Simple Summary:**

Sugarcane ratooning ability is directly related to sugarcane production costs and planting benefits. There are several questions within the field that we have explored in this review and that remain to be answered. What is the genetic basis of ratooning ability? How do these traits form and evolve? How does the environment affect the ratooning ability? Where should the research focus of sugarcane ratooning ability be placed in the future? How can technical methods be optimized for breeding sugarcane with strong ratooning ability? In this paper, we reviewed previous studies in terms of the definition, phenotypic traits, major influencing factors, genetic basis, and the physiology associated with sugarcane ratooning ability. We also highlighted the shortcomings of existing research on ratooning ability and suggested the focuses of future studies.

**Abstract:**

Sugarcane is an important sugar crop and it can be subjected to ratooning for several years. The advantages of ratooning include quality improvement, efficiency enhancement, and reduced costs and energy use. The genotype, environment, cultivation management, and harvesting technology affect the productivity and longevity of ratoon cane, with the genetic basis being the most critical factor. However, the majority of research has been focused on only limited genotypes, and a few studies have evaluated up to 100 sugarcane germplasm resources. They mainly focus on the comparison among different genotypes or among plant cane, different selection strategies for the first and second ratoon crops, together with screening indicators for the selection of stronger ratooning ability. In this paper, previous studies are reviewed in order to analyze the importance of sugarcane ratooning, the indicative traits used to evaluate ratooning ability, the major factors influencing the productivity and longevity of ratooning, the genetic basis of variation in ratooning ability, and the underlying mechanisms. Furthermore, the shortcomings of the existing research on sugarcane ratooning are highlighted. We then discuss the focus of future ratoon sugarcane research and the technical methods that will shorten the selection cycle and increase the genetic gain of ratooning ability, particularly the development of linked markers. This review is expected to provide a reference for understanding the mechanisms underlying the formation of ratooning ability and for breeding sugarcane varieties with a strong ratooning ability.

## 1. Introduction

Sugarcane (*Saccharum* spp. hybrids) is an important sugar crop that can be subjected to ratooning over multiple years. Sucrose from sugarcane accounts for 86% of the world’s [1] and 90% of China’s total sugar output [2]. In China, approximately 60–70% of sugar production costs are spent on raw sugarcane stalks. Compared with newly planted sugarcane, i.e., plant cane, ratoon cane has multiple advantages including faster leaf spreading, more rapid plant growth, earlier strike maturity, and reduced production costs due to savings on fertilizers, seed cane, field preparation, planting and early field management.

Sugarcane stalks are a fresh agricultural product that must be processed as soon as possible after harvesting. The immediate processing is performed to minimize the conversion of sucrose into reducing sugars within the sugarcane stem to increase the sugar output. Sugarcane originated from the tropics and requires a warm climate, and only when the temperature is above 20 °C can the mean effective accumulated temperature meet the requirement for sugarcane normal growth and development. Previous data also suggest that the non-optimum germination or sprouting temperatures, too low or too high, may be a factor for yield decline in ratoon cane [3]. Compared with plant cane, ratoon plants have an established, and strong root system, representing the unique skeleton of carbon and energy source for the initial plant development [4]. The root system, which is essential for regrowth of sugarcane and the ratoon vigor of each cycle [4], can be used for water transport to leaves during the period of photosynthesis, in which photosynthetic products are accumulated and in turn promote a rapid leaf expansion and plant growth during the early growing stage. Therefore, ratoon plants have more effective accumulated temperature and longer effective growth period, resulting in more sugar accumulation and earlier technical maturity. In contrast, newly planted sugarcane plants firstly need to grow roots, especially permanent roots, which requires a relatively longer period and a higher effective accumulated temperature. As a result, the newly planted sugarcane does not use light and thermal sources efficiently at this stage. Therefore, from the perspective of energy utilization, ratoon sugarcane has a significant energy-saving characteristic. According to published reports, ratoon sugarcane requires only 89,040,000 calories per ton of sugarcane production, while newly planted sugarcane requires 204,550,000 calories per ton [5], suggesting that plant cane uses 2.3 times more calories than ratoon cane. This opinion is supported by Hunsigi and Krishna (1998) [6], who believed that an irrigated ratoon crop requires only 295 days for its maturity compared with 482 days in plant cane.

Ratoon cane is very important in sugarcane production. After the harvest of sugarcane, the underground portion of the strikes gives rise to a succeeding crop, which is known as ratoon crop. Sugarcane ratooning is a planting system that is generally adopted by each sugarcane-producing country. However, the number of ratoons varies from 1–8 [7] (Table 1). The proportion of the ratoon cane is generally around 50% of the cultivated area, and can even reach 75% in some regions (Table 1). The average proportion is 50–55% in tropical areas, while approximately 40–45% in subtropical areas [8].

The cost of sugarcane production is much higher in China than in other countries including Brazil. Except for the low cost of arable land, better ecological and soil conditions, and the complete mechanical operations in sugarcane production, sugarcane variety with a strong strike is one of the most important reasons for the low cost in Brazil. In the Brazil agricultural practice, a cycle of consecutive ratooning for 4–5 years after the harvest of plant cane is generally adopted due to the standard cycle of plant-cane followed by three years of ratooning not being profitable. In India, it has been reported that the cost of ratoon crop is 25–30% lower than that of plant cane [7]. However, due to low yields (40–50 t/ha), ratoon crop accounts for only 40% [8,9] or 40–45% [7] of the total cane area and sugarcane is only ratooned for one to two years in India [10], resulting in the relatively higher cost in sugarcane production. This is supported by another report, which suggests that ratoon cane contributes only 30% of the total cane production, though it accounts for over 50% of the acreage [11]. Another report [7] also pointed out the problem of low yields of ratoon crop, indicating only 30–35 t/ha as compared to 65–75 t/ha of plant cane in India. In Pakistan, ratoon crop can save 25–30% in past [12] and 30–40% in current production costs [13], indicating a widening gap in costs. In China, there is a high proportion of ratoon cane. In Guangxi and Yunnan, the two most important sugarcane-producing provinces accounting for more than 80% of the total areas in China, ratoon crop accounts for 50–60% and 70% of the sugarcane production, respectively. However, due to the poor ratooning ability of the varieties, a cropping system of plant cane followed by two ratoons is generally adopted except for Zhanjiang Guangdong, where only one ratoon is adopted mostly due to the serious pests and smut caused by *Sporisorium scitamineum*. Therefore, the short longevity of ratooning is considered to be the major cause of high sugarcane production costs in China.

**Table 1:** Comparison of ratoon status in major sugarcane planting countries.

Country Name	Ratoon Percentage (%)	Ratoon Age (Year)	References
America	80–85	2–3	[14]
Brazil	80–90	4–5	[14,15]
Australia	80–85	2–3	[14]
South Africa	80–90	4–5	[14,15]
China	50–70	2–3	[15]
India	>50	1–2	[7,8,9,10]
World	75	4–7	[8,14,15]

From all the above, ratoon crop reduces production costs and benefits growth through energy saving by the reduction of inputs and utilization of residual manure and moisture. With the rising labor costs, the gap in cost between ratooning and replanting will further be widened. Additionally, ratooning is undoubtedly a simple and easy way to improve the efficiency of sugarcane production. However, the yields of ratoon cane decline with age. In this paper, we review the achievements of sugarcane ratooning research, highlight shortcomings, and propose research ideas. We hope that this review enhances the understanding of the research progress of ratooning ability, and is beneficial to develop sugarcane variety with strong ratooning ability.

## 2. Definition of Sugarcane Ratooning Ability

It is widely accepted that variations in ratooning ability exist in different genotypes and hybrid offspring [16]. The definitions of ratooning ability that have been provided by different authors are as follows: the yield of second ratoon as a percentage of the yield of newly planted sugarcane [17]; ratoon crop performance as a percentage of a reference yield, usually that of the plant cane, first ratoon or the mean of these two crops [18]; the ability to maintain yield with the increased ratooning years [19]; the yield of the ratooning year as a percentage of the yield for the reference variety of that year [20]; a joint evaluation based on the quantity, growth speed, strength, final stem formation rate, number of effective stems, and yield of ratoon cane in the last season [21]. Although there are differences in description of ratooning ability, the core is similar, that is, a good performance in ratoon cane yield or ratoon can produce several profitable crops. In other words, the longer the ratooning cycle and the smaller yield decline in ratoon crops, the stronger ratooning ability.

## 3. Phenotypes of Ratooning Ability in Sugarcane

Ratooning increases the income of sugarcane growers due to the saving cost in cultivation, and increases the income of industry because of mature earlier, better juice quality and thus improves sugar recovery at times of the crushing season compared with plant cane [7,13]. For example, in plant cane and the second ratoon, the average sucrose content was 14.84% and 16.54%, respectively [22]. Most studies on sugarcane ratooning ability have focused on analyzing the variation in ratooning ability based on phenotypic traits [5,17,19,20,23,24,25,26,27,28,29]. Generally, the most effective way for the improvement of sugarcane ratooning ability is to select lines directly based on the yield performance of ratoon crops. However, it is not conducive to shortening the selection cycle, and the huge segregated population in sugarcane hybrid F_1_ limits this measure due to considerable time and resources. For example, to identify one commercial quality variety from the original F_1_ population requires 11 years of sequentially planted selection from approximately 75,000 genotypes [17]. An alternative approach is to select lines based on the yields of plant cane because varieties with high plant cane yields normally produce high ratoon crop yields [24,30]. Indirectly selecting genotypes with strong resistance to diseases and insect pests may also increase the ratooning ability of the selected sugarcane breeding materials [17,31]. In some cases, the ratooning ability has been indirectly evaluated by assessing the biomass or light utilization efficiency of sugarcane [30], and assessing drought tolerance in those arid or semi-arid cultivated regions is also suggested [32].

Ratooning ability is a trait that a commercial quality variety must have. Indicative traits of a strong ratooning ability include both morphological indicators of sugarcane root residue/stubble and traits that directly contribute to cane yield and sugar output, such as a high number of stalks, high viability of buds, large number of viable buds, large number of viable roots, high cane yield, high sugar output [5,17,19,24,25,28,29,33], and high tillering rate in plant cane [34]. Milligan et al. found that stalk number was the only trait in the plant cane markedly correlated to the yield of the ratoon crop [17]. Qin et al. demonstrated in their research that sugarcane lines with strong ratooning ability displayed rapid germination, higher germination rate and tillering rate, and higher stalk number in plant cane [28,35,36]. Additionally, a higher stubble germination rate and the larger shoot number were observed in the ratoon crops, which result in high stalk number and higher cane yields than those in plant-cane. A similar observation was obtained by other reports [11,14,33]. It is also believed that the ratooning ability of sugarcane is mainly identified by four important factors, namely, root traits, the total number of strikes or shoot population, stalk number, and cane yield. Good performance on the four aspects above in its plant cane and the ratoon crops is necessary for the selection of varieties with a strong ratooning ability. In addition, a sugarcane variety is considered to have a poor ratooning ability if the cane yield in ratoon crop is lower than that of its plant cane. The morphological characteristics of sugarcane stubble are closely related to the ratooning ability of the sugarcane [29]. In addition, sugarcane varieties with strong ratooning ability have a low stubble mortality rate and a short internodal length of underground stems, together with the obviously larger total number of underground buds and the effective tillers [29]. Generally, if there is an increased number of effective tillers formed by the lower buds of the main stems, and there is an increased total number of effective tillers on the main stems, then the variety likely has strong ratooning ability [29].

There was a significant interaction effect between varieties and growing seasons for all yield and qualitative traits except for the purity of sugarcane juice [19]. In addition, in the second ratoon crop, both cane yield and total recoverable sugars (TRS) were significantly higher in the varieties with strong ratooning ability than in those with weak ratooning ability. Based on an investigation of later crop, Olaoye found that single stalk weight, cane yield, total soluble solids (Brix), and sucrose percentage, were highly heritable traits that displayed the potential to obtain high genetic gain [20]. Additionally, a study on the genetic relationships among sugarcane traits in a large population indicates that stalk number was the primary determinant of cane yield and thus became more important trait in determining cane yield in the ratoon crops (r = 0.77), much higher than those of stalk diameter (r = 0.52) and stalk length (r = 0.33) [33]. Research also indicated that, for varieties with poor ratooning ability, the ratoon crops had a much lower cane yield than the plant-cane [29,34] or a sharp decline in cane yield in the first ratoon compared with plant cane [18]. Meanwhile, the yield decrease was only observed in varieties with strong ratooning ability in the second ratoon crop [20]; however, it is desirable to perform ratooning in sugarcane production for as many years as possible. In addition, a significant correlation was found between the number of shoots and the genotype/cane yield/harvesting time in sugarcane [37]. In addition, the relationship between the ratooning ability and the changes in endogenous hormone contents during germination of underground buds in stubbles have also been studied [5]. Recent studies have also indicated that the experimental location is particularly important for evaluation of the ratooning ability [38].

In brief, for the selection of ratooning ability, direct indicators are the stubble morphology, stalk number, and the germination and tillering rates in the plant cane and the ratoon crops, while indirect indicators included disease resistance especially smut, pest resistance, biomass, light use efficiency, and hormone content during stubble bud germination. The number of indicators used in selection may vary, but researchers have the same or a similar opinion on those indicators. In addition, more attention should be paid to the selection of the experimental location, mostly due to the reason that the effect of the location on ratooning ability is visible.

## 4. Main Factors Influencing Longevity and Productivity of Ratoon Sugarcane

The ratooning ability or good ratooning potential is an essential pre-requisite or the most critical factor for good ratoon [11,38,39]. A series of investigations have been conducted on the factors that affect the longevity of ratoon cane. The genotype, cultivation management, and environment contribute to the ratoon crop in descending order [4,11]. The ratoon crop yields decline typically with age [39]. Studies have also shown that, in subtropical regions, a major bottleneck for improving ratoon productivity is the poor germination rate of buds in the stubble remaining after winter harvesting [8]. There is a report that the poor germination rate of the ratoon crop not only affects the number of seedlings per unit area, but also leads to a large number of ineffective tillers throughout the growing season, resulting in the lower stalk number at harvest [40]. The trait of stalk number has the greatest impact on sugarcane yield. Guangxi, the largest sugarcane producing area accounting for more than 60% of the total sugar in China, is located in the subtropical region, where germination is a severe challenge during ratooning due to the serious smut infection during growing process and the frequent rainfall during harvest. The breeding and promotion of sugarcane varieties with strong ratooning ability is a technical approach to effectively solve this problem. This is because the ratooning ability directly affects the germination rate of the ratoon crop, thereby directly influencing the establishment of the high-yielding seedling population, and ultimately cane yield. Therefore, the ratooning ability is one of the most important target traits in sugarcane breeding and has always been valued by breeders [8,19,23,26,27,36,41].

From both the perspective of reducing production costs and improving the productivity of the ratoon crops, breeding and growing varieties with a strong ratooning ability is the most important prerequisite for extending the number of ratooning years and increasing the yield of the ratoon crops. Furthermore, in sugarcane-producing areas with low temperatures, frost, drought, pests, diseases (especially smut), stem borers, or extensive management, the ratooning ability of sugarcane varieties is particularly important for extending the number of ratooning years and increasing the yield of ratoon crops. 

## 5. Genetic Research on the Variation in Ratooning Ability between Different Sugarcane Genotypes

Sugarcane genotypes with higher proportions of the genetic background of *Saccharum spontaneum* display stronger ratooning ability [42] because the characteristics of a species can be affected by kinship [43], i.e., hereditary basis. Sugarcane ‘nobilization’ breeding aimed at bringing the genes controlling vigor, vitality, stress resistance, and strong ratooning ability from wild species into original cultivated species, i.e., ‘noble’ *S. officinarum*. A wild species *S. spontaneum,* the mostly used and studied, was the first species to naturally hybridize with *S. officinarum* [44]. Other wild species that have desirable traits and have been used in sugarcane breeding are *S. rubustum* and species in closely related genera including *Erianthus arundinaceum* and *Narenga porphyrocoma* [45,46,47,48]. Of these, *N. porphyrocoma* was firstly utilized in China in recent years to improve the ratooning ability and stress resistance, especially smut resistance of sugarcane varieties [49]. The combining ability and heritability of traits including ratooning ability have been investigated in the genetics and breeding of sugarcane. The combining ability refers to the ability of parental traits to be combined in hybrid crosses, which is an important basis for selecting hybrid parents and making cross combinations. Studies have shown that the ratooning ability is jointly affected by the general and specific combining abilities of both the male and female parents [42]. The inheritance of sugarcane ratooning ability and the relationship of traits between plant cane and ratoon crops has been well examined by Milligan et al. [17], which suggests that the genetic coefficient of variation of ratooning ability is largest for cane yield and sucrose yield, and the selection for stalk number in the younger crops will increase the yields of older crops.

The ratooning ability was found to vary among 138 exotic sugarcane germplasm accessions [50]. This variation provided a genetic basis and potential for the breeding of sugarcane varieties with strong ratooning ability. At present, the selection of ratooning ability for breeding is based on the ratoon crops and plant crop. One earlier study showed that selecting in the ratoon crop, rather than in the plant cane, is more conducive to obtaining sugarcane lines/clones with a strong ratooning ability [51]. The offspring derived from 45 cross combinations made using 10 ROC series parents, including ROC22, were investigated for the ratooning ability [39]. Regardless of whether these parents were used as the male or female, the parent’s ratooning ability had a greater effect on the ratoon crops than on the plant cane in their offspring. Furthermore, the heritability of the ratooning ability is higher for the ratoon crops than for the plant cane. In addition, Zhou et al. proposed that it is possible to select a genotype with both high yield and strong ratooning ability by covariance analysis [52]. Researchers demonstrated that there was a close correlation between the stalk count and the ratooning ability in sugarcane. Meanwhile, the ratooning ability was negatively correlated with single stalk weight and commercial cane sugar (CCS). Therefore, strengthening CCS through selection without considering the ratooning ability is not conducive to pyramiding the genotypes with strong ratooning ability [44].

## 6. Mechanism Underlying the Variation in Sugarcane Ratooning Ability

Morphological basis. Previous studies on the morphology of sugarcane stubble reached the unanimous conclusion that strong ratooning ability-related stubble has deep roots (long roots), a large number of buds and live buds, and a large number of permanent roots [5,9,23,29,36], along with a rational leaf size and the reasonable tillering ability [8]. However, no comprehensive and quantitative morphological analysis has been reported for sugarcane root growth and development using modern technologies and equipment.

Physiological basis. Sugarcane can concentrate CO_2_ around Rubisco and utilize the NADP malic enzyme type of C4 photosynthesis, thus exhibiting superior photosynthesis over C3 plants [53,54]. Among the photosynthetic parameters, the chlorophyll fluorescence and stomatal conductance of the leaves of the ratoon cane are significantly correlated with ratoon sugarcane yield [10]. Decreased nitrate reductase activity and cation exchange capacity at the root–soil interface were found to cause a decline in dry matter accumulation, which ultimately resulted in reduced ratoon sugarcane yield [11]. Chlorophyll content and chlorophyll fluorescence have also been shown to affect ratoon sugarcane yield [9]. In addition, during bud germination in ratoon crops, the endogenous abscisic acid (ABA) content was significantly higher in lines with strong ratooning ability than in lines with weak ratooning ability [33]. The stronger the ratooning ability, the higher the ABA content, while the lower the auxin (IAA)/ABA and gibberellic acid (GA_3_)/ABA content ratios, the higher the ratooning ability, however the IAA, cytokinin (CTK), and GA_3_ contents were not significantly correlated with ratooning ability [33]. It was observed that ethephon, IAA, and CTK played a role in the germination and growth of sugarcane lateral buds [55,56]. 

Molecular basis. There has been little research on the molecular basis of ratooning ability variation. ABA was found to regulate the withering of ineffective tillers through hormonal interactions in the form of molecular signals [55,56]. Overexpression of the *TB1* gene in a sugarcane line resulted in a reduced tiller number [57]. So far, no report has been published on the molecular mechanism underlying the ratooning ability of sugarcane. However, related research has been conducted in rice, which is also monocotyledonous and has tillering traits [58]. This research in rice has provided a reference for the study of the molecular mechanism responsible for the variation in sugarcane ratooning ability.

Differences in rhizosphere microorganisms. Plant-soil organisms, especially microorganism interactions, play important roles in crop health, crop yield, and soil quality. However, the relationship between the diversity of rhizosphere microorganisms and the ratooning ability is still unclear. In this field, most studies have focused on the investigation of the differences of soil physicochemical properties, soil nutrient, and change of rhizosphere microorganism between plant cane and ratoon crop, or among the different practices of cultivation management [59,60,61,62], including residue mulching [63], plant growth regulators [64], bioagents [65], and rotation [61,66]. Lin et al. found 38 differentially expressed proteins in rhizospheric soil of ratoon crop compared with plant cane [60]. Recently, significant differences in the diversity of rhizosphere bacteria have been found between sugarcane varieties [67,68]. 

## 7. Shortcomings of Existing Research

Modern sugarcane varieties (*Saccharum* spp. hybrids) are interspecific hybrids and the allopolyploids derived from the hybrids of homopolyploids, and may have up to 120 chromosomes. In fact, the number of chromosomes can be different between genotypes, for example, from 106 to 111 among 10 main sugarcane cultivars in China [69]. Of these, 70–80% of the chromosomes are from a tropical species (*S. officinarum*) that has 80 chromosomes (octaploid, 2n = 8×) and 10–20% are from wild species *S. spontaneum* that has 40–128 chromosomes (2n = 4× to 12×) [70,71]. Recombinants between the two species account for 10% of the genome in modern varieties [72]. It is precisely because of this highly heterozygous genetic background that the offspring of sugarcane hybrids are widely segregated and the probability of aggregation of excellent traits is extremely low (1/100,000–1/300,000). Therefore, for a long time, sugarcane cross breeding had to rely on large segregating populations. In China alone, in spite of more than one million F1 seedlings being planted in the field each year, a commercial cultivar with high yield, disease resistance, especially primary diseases including smut resistance, and strong ratooning ability has not been yet developed. Approximately 95–97% of planted F_1_ seedlings are discarded after observation in the first year, without ratooning.

Sugarcane breeding in China has made great progress. The new varieties Liucheng 05–136, Guitang 42, and Yuetang 93–159 have accounted for 65–70% of the production. ROC22 is a variety that has been widely used in production for over fifteen years due to its broad adaptability. However, ROC22 shows serious loss in the field in recent years due to smut. This situation is more serious for the ratoon crops. As a result, the yield of the first ratoon crop is lower than that of the plant cane. Due to this problem, the proportion of ROC22 in production has fallen from 85% to less than 20% in 2020. Liucheng 05–136 and Guitang 42 are replacing ROC22, and account for over 50% of production. However, these two new varieties still display poor resistance to smut. Thus, there is more than a 30% incidence of smut in the first ratoon crop. Therefore, in China, the problem of the short ratooning longevity of the leading sugarcane varieties needs to be solved. Although selection based on the ratooning population is helpful for improving ratooning ability, especially in the second ratoon [17], the cost and occupation of arable land need to be considered, and both questions are not easily addressed.

Sugarcane cross breeding relies on a huge segregated population. There is still a lack of effective and high-throughput selection technology suitable for early segregating generations and large populations. Although the selection of ratooning ability based on phenotype is intuitive and effective in general, it is still difficult to identify and select varieties with strong ratooning ability, disease resistance, and high yield simultaneously. However, we can consider and select those traits with high heritability and associated with the three breeding target traits mentioned above, such as tillering ability for strong ratooning ability, high smut resistance for disease resistance, and a high number of millable stalks for high yield. 

## 8. Research Prospects

**Figure 1:**
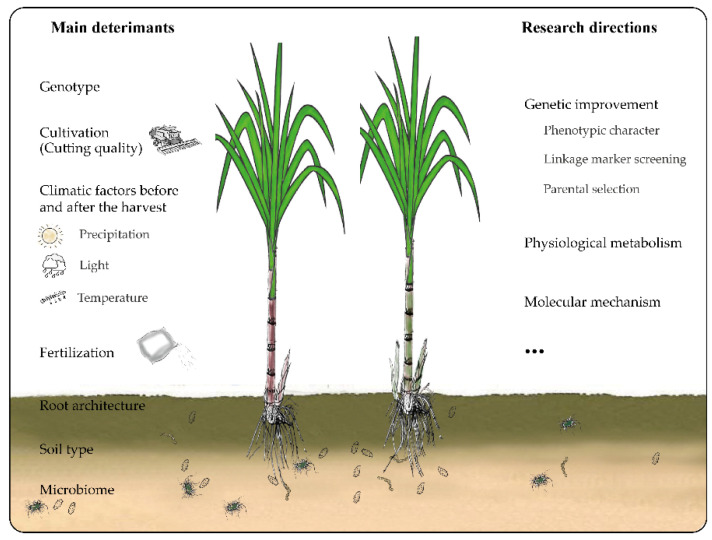
The main determinants and research directions for sugarcane rationing.

The ratooning ability of sugarcane is not affected by a single factor, but by multiple factors including genotype, environment (soil, temperature, humidity, and water supply in the growing area), and cultivation technologies (Figure 1). Optimizing the sugar production per unit area is the ultimate goal of breeding sugarcane varieties with strong ratooning ability. Therefore, research on sugarcane ratooning ability should be integrated into the breeding of varieties with strong ratooning ability. Regarding the commercial cultivation, breeders need to consider at least three traits, namely, ratooning ability, main disease resistance in production (such as smut resistance in China), and sugar production per unit area. To select these traits during breeding, it is necessary to identify and develop markers associated with the traits. High-throughput technologies should then be used to analyze the populations.

Markers associated with target traits are valuable for breeding. In sugarcane, markers associated with sugarcane traits, including sucrose content and sugar content, have previously been studied [73,74,75,76]. In addition, markers associated with sugarcane yellow spot resistance have also been studied [77]. However, thus far, only markers associated with the major gene, *Bru1*, which is responsible for sugarcane brown rust resistance, have been used in breeding. The first marker to be identified was 10 cM from the *Bru1* gene [78]. Furthermore, markers that were 1.9 cM and 2.2 cM from the gene were observed [79], but only the markers that were 0.28 cM and 0.14 cM from the *Bru1* gene were identified [80] and used to assist the selection in breeding [81,82,83]. Nevertheless, these markers still have the problem of false positive [81], that is, the markers can be detected in a line without the actual disease resistance. It is difficult to identify markers closely associated with target traits because a complete reference genome is still unavailable for sugarcane; however, some progress has been made [84,85,86,87]. Therefore, adding the full-length transcriptome unigene data of the hybrid parents as a supplement to the reference genome sequence, and using these data in biparental F_1_ population mapping, may be beneficial for identifying target trait-associated genes. What should also be stressed here is that mining key regulatory genes associated with excellent traits, clarifying the allelic variation of these key genes, and analyzing the distribution and genetic effect of haplotypes, are all important for elucidating sugarcane ratooning ability and for gene editing breeding methods.

There are several technical approaches that are used to identify associated markers and genes. First, polymorphic data of the whole genome obtained by Pool-seq can be compared to the corrected transcriptome unigene database to identify the key genes associated with sugarcane ratooning ability. This is a cost-effective strategy and needs to be considered. Second, a biparental F_1_ population and sugarcane single nucleotide polymorphism (SNP) chip can be used to perform genotyping and marker analysis. Successful cases using this approach have previously been reported [88,89,90,91,92]. However, the actual application value of the obtained quantitative trait loci (QTL) needs further verification. A third approach involves the utilization of inbred populations, such as R570 inbred populations [78,79]. This approach helps to tackle the fact that modern sugarcane varieties are highly heterogeneous, and even selfing offspring are still highly heterozygous, and thus the target traits are segregating. A fourth approach, which has been successfully applied in sugarcane [93,94], involves the utilization of natural populations and the simplification of whole genome sequencing technology. 

## 9. Conclusions

Ratooning can largely reduce production costs compared with replanting sugarcane. Labor costs increase yearly, and the cost difference between ratooning and replanting sugarcane widens. In this paper, previous studies on sugarcane ratooning ability were reviewed in terms of the definition, phenotypic traits, major influencing factors, genetic basis, and the formation mechanisms. In addition, the shortcomings of existing research on ratooning ability were highlighted and the focuses of future studies were suggested. We do hope that this review can provide a reference for understanding the mechanisms underlying sugarcane ratooning ability, and for breeding sugarcane varieties with strong ratooning ability.

## Data Availability

Not applicable.
